# Study on the Hepatoprotection of *Schisandra chinensis* Caulis Polysaccharides in Nonalcoholic Fatty Liver Disease in Rats Based on Metabolomics

**DOI:** 10.3389/fphar.2021.727636

**Published:** 2021-09-21

**Authors:** Yanbo Feng, Han Li, Cong Chen, Hao Lin, Guangyu Xu, He Li, Chunmei Wang, Jianguang Chen, Jinghui Sun

**Affiliations:** College of Pharmacy, Beihua University, Jilin, China

**Keywords:** *Schisandra chinensis* Caulis, polysaccharides, nonalcoholic fatty liver disease, metabolomics, UGP2, UGDH

## Abstract

The aim of this study was to investigate the hepatoprotection of *Schisandra chinensis* Caulis polysaccharides (SCPs) in the nonalcoholic fatty liver disease (NAFLD) induced by high-fat diet (HFD) in rats. A total of 30 Wistar rats were randomly divided into the control group (CON), model group (MOD), and *Schisandra chinensis* caulis polysaccharide (SCP) group. Except for those in the CON group, the other rats were fed with high-fat diet for 4 weeks to establish an NAFLD model. From the 5th week, rats in the SCP group were given SCP solution (100 mg kg^−1^) by gavage for 6 weeks, and those in the CON and MOD groups were given an equal volume of distilled water in the same way. Aspartate aminotransferase (AST), alanine aminotransferase (ALT), triglyceride (TG), total cholesterol (TC), low-density lipoprotein cholesterol (LDL-C), high-density lipoprotein cholesterol (HDL-C) levels in serum, the malondialdehyde (MDA) level, glutathione peroxidase (GSH-Px), and superoxide dismutase (SOD) activities in the liver tissue were detected. The small molecular metabolites in the blood of rats were determined by the metabolomics method of ultra-high-performance liquid chromatography-quadrupole/electrostatic field orbitrap high-resolution mass spectrometry (UHPLC-Q-Orbitrap-MS/MS) combined with multivariate analysis. The enrichment analysis and pathway analysis of the different metabolites were carried out. The therapeutic mechanism of SCP in NAFLD rats was verified by western blot. The results showed that the levels of AST, ALT, TG, TC, and LDL-C in the serum of rats in the SCP group were significantly lower, and the levels of HDL-C were significantly higher than those in the MOD group. The screening and analysis of the metabolic pathways showed that SCP could alleviate the development of NAFLD by regulating the expression of UDP-glucose pyrophosphorylase (UGP2), UDP-glucose 6-dehydrogenase (UGDH), acetyl CoA carboxylase (ACC), and fatty acid synthase (FAS) in the liver of NAFLD rats. This study may provide a theoretical basis for the development and utilization of SCP.

## Introduction

Nonalcoholic fatty liver disease (NAFLD) refers to the accumulation of fat in the liver without excessive drinking or other known liver diseases and gradually evolved into nonalcoholic steatohepatitis, liver cirrhosis, and hepatocellular carcinoma (Abdelmoneim et al., 2021). With the improvement of people's living standards, excessive intake of sugar and high-fat and high-calorie diets can easily lead to increased blood lipids and fatty liver disease. In recent years, the prevalence of NAFLD in the world has been on the rise (Schön, 2021), so it is of great significance to study the body changes under the conditions of nonalcoholic fatty liver disease.

*Schisandra chinensis*, the dry and mature fruit of a *Magnoliaceae* plant *Schisandra chinensis* (Turcz.) Baill., is widely used because of its significant hepatoprotection, hypolipidemia, and antioxidation (Chen et al., 2019; Zhu et al., 2019; Yan et al., 2020). However, other parts of *Schisandra chinensis*, such as caulis, have not been utilized. According to statistics, nearly 1,000 tons of *Schisandra chinensis* caulis are pruned and discarded every year only in Northeast China where *Schisandra chinensis* is planted, resulting in a waste of resources (Mocan et al., 2016). Studies have shown that the components in the dried caulis of *Schisandra chinensis* are similar to those in its fruit, with a significant hypolipidemic effect (Zheng et al., 2014; Li et al., 2018). Therefore, it is speculated that *Schisandra chinensis* fruit and *Schisandra chinensis* caulis have the same protective effect against fatty liver injury, but the specific mechanism is not clear.

All the effects of exogenous substances, pathophysiological changes, or genetic variation will be reflected in various biological pathways, which will interfere with the steady-state balance of endogenous metabolites to change the concentration and proportion of various substances in endogenous metabolites, and at the same time, the metabolites will interact with upstream genes and proteins to feed back it them the upstream life activity network, revealing the life activity picture of the body at the overall level (Bjerrum et al., 2017; Li et al., 2021). Metabolomics is to study the regulation and response of organisms to the changes of internal and external environments from the perspective of the system so as to expand the research of the disease mechanism from the internal differences of organisms to the interaction between organisms and their environment, making the research more systematic, dynamic, and accurate (Bjerrum and Nielsen, 2019). Therefore, in this study, a large number of endogenous metabolites in the blood of NAFLD rats were detected qualitatively and quantitatively before and after the administration of the *Schisandra chinensis* caulis polysaccharide (SCP) by the metabolomics method based on UHPLC-Q-Orbitrap-MS/MS combined with multivariate analysis, and its specific mechanism was further explored.

## Materials and Methods

### Chemicals and Reagents

Dried *Schisandra chinensis* caulis (Ji’an *Schisandra Chinensis* Planting Base in Jilin Province, Jilin, China); ALT, AST, TG, TC, HDL-C, LDL-C, MDA, GSH-Px, and SOD test kits (Nanjing Jiancheng Bioengineering Research Institute, Nanjing, China; batch number: 20191212, 20191212, 20191214, 20191212, 20191214, 20191212, 20210721, 20210721, and 20210721, respectively); and UGP-2, UGDH, FAS, and ACC antibodies (Wuhan Abclonal, Wuhan, China) were the chemicals and reagents.

### Extraction and Determination of the *Schisandra chinensis* Caulis Polysaccharide

The dried *Schisandra chinensis* caulis was ground into powders, and then, the powders were immersed in 10 times the volume of distilled water at room temperature overnight. The next day, the mixture was boiled at 100°C for 3 h to obtain the water extraction, then the water extraction was concentrated at 80°C by rotary evaporation, and 95% ethanol was added to the supernatant. Finally, the ethanol concentration was adjusted to 75%, and then, the mixture was precipitated at room temperature overnight. The precipitate was collected by centrifuging it at 20°C for 15 min, then washed with 95% ethanol and anhydrous ethanol one time, and freeze-dried to obtain the powdery SCP.

The total carbohydrate content of SCP was determined by the phenol sulfuric acid method with glucose as the standard. The monosaccharide composition and molecular weight distribution of SCP were determined by high-performance liquid chromatography (HPLC).

### Animal Grouping and Administration

Male Wistar rats, weighing 250–300 g, were provided by Changchun Yisi Experimental Animal Technology Co., Ltd. (Changchun, China), and the certificate number was SCXK (Ji) 2019-0007. The rats were reared in separate cages in a sterile feeding room at a temperature of 18–23°C and in a humidity of 40–60%. The standard feed and the high-fat feed for experimental rats were provided by Changchun Yisi Experimental Animal Technology Co., Ltd. (Changchun, China). The high-fat diet contained lard (15%), sucrose (20%), cholesterol (1.2%), sodium cholate (0.2%), casein (10%), calcium hydrogen phosphate (0.6%), and basic diet (53%). Animal experiments were approved by the Experimental Animal Ethics Committee of Beihua University (Jilin, China), and all the experimental procedures were performed in accordance with the Guide for the Care and Use of Laboratory Animals.

**FIGURE 1:**
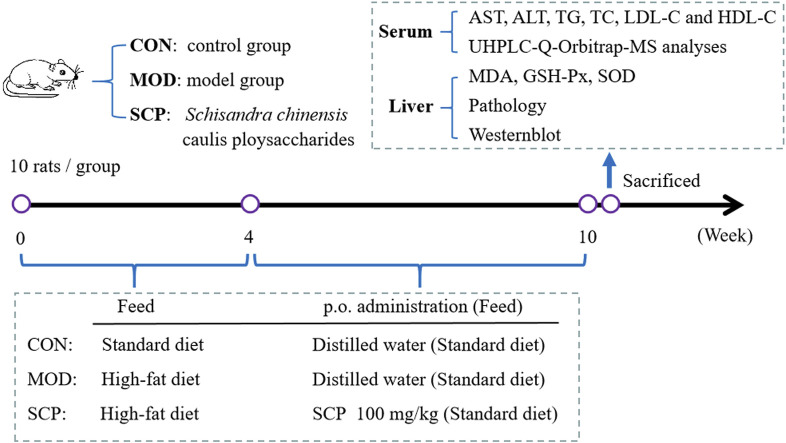
Grouping and administration of rats and experimental processes.

A total of 30 rats were randomly divided into the control group (CON), model group (MOD), and *Schisandra chinensis* caulis polysaccharide (SCP) group. Except for those in the CON group, all rats were fed with the high-fat diet for 4 weeks. Then, rats in the SCP group were given 100 mg kg^−1^ SCP solution for 6 weeks by gavage from the 5th week, and those in the CON and MOD groups were given an equal volume of distilled water in the same way. Rats in all groups were given standard diet from the 5th week to the 10th week. Blood samples of all the rats were collected from the abdominal aorta of anesthetized rats by the intraperitoneal injection of 25% urethane (100 mg kg^−1^) on the 11th week. The blood samples were left standing at room temperature for 1 h and then centrifuged at 3,000 r min^−1^ to separate the serum, and the serum samples were stored at −20°C for standby. The hepatic tissue was washed with cold saline and divided into three parts: the first part was fixed with 10% neutral formaldehyde for histopathological examination, the second part was prepared into homogenates for the detection of antioxidant indexes, and the third part was preserved at −80°C for western blot analysis. The grouping and administration of rats and experimental processes are shown in Figure 1.

### Histomorphological Observation

The liver of rats was separated, the redundant tissue and fascia attached on the surface of liver were stripped, and the liver tissue was fixed with 10% formalin solution at room temperature for 24 h. The liver tissue was routinely sliced (5 μm thick), and the slices were embedded in paraffin and stained with Hematoxylin-eosin at room temperature for 10 min. Pathological changes of the liver tissue were observed under a light microscope (magnification:×200, ×400).

### Determination of Biochemical Indexes

The levels of serum aspartate aminotransferase (AST), alanine aminotransferase (ALT), triglyceride (TG), total cholesterol (TC), low-density lipoprotein cholesterol (LDL-C), high-density lipoprotein cholesterol (HDL-C), liver malondialdehyde (MDA), glutathione peroxidase (GSH-Px), and superoxide dismutase (SOD) were detected by following the instructions of the test kits.

### Metabolomic Analysis

One hundred µl of the serum was added with 400 µl of methanol. The serum–methanol solution was mixed using a vortex oscillator for 20 s and then centrifuged at 12,000 rpm and 4°C for 5 min to obtain the supernatant. The supernatant was evaporated to dryness with nitrogen, and its volume was fixed to 1 ml with methanol for use.

An Ultimate 3000 ultra-high-performance liquid chromatography (UHPLC) system (Thermo, San Jose, CA, United States) was used for the separation, in which the chromatographic column was a Supelco C18 column (3.0 × 50 mm, 2.7 μm; Sigma-Aldrich, United States), the column temperature was 35°C, and acetonitrile and water with 0.1% formic acid were used as mobile phases A and B, respectively; the gradient elution procedures were as follows: 65% B (0–5 min), 65–45% B (5–10 min), 45–15% B (10–20 min), 15–10% B (20–25 min), and 10–65% B (25–30 min); the flow rate was 0.3 ml/min; and the injection volume was 10 μl. The UHPLC system was connected with a mass spectrometer.

Q-Orbitrap-MS/MS (Thermo, San Jose, CA, United States) was used for the determination by mass spectrometry, in which the positive ion mode and negative ion mode were set. The electrospray ionization (ESI) source conditions were as follows: the sheath gas flow was 35 Arb, the auxiliary gas flow rate was 10 Arb, and the sweep gas flow was 1 Arb. The S-Lens RF was 50%, the capillary voltage was set to +4.0 kV, the capillary temperature was 340°C, and the mass scanning range was *m/z* 100–1,500 Da. LC-MS data were extracted, filtered, and normalized using Thermo software Xcalibur (version 4.3) to obtain the molecular weight, retention time, and absorption peak area of the compounds in each sample.

SIMCA 14.1 software (Umetrics, Kinelon, NJ, United States) was used for the multivariate data analysis, and potential marker compounds with importance of variables (VIP) >1 were screened by using principal component analysis (PCA), orthogonal partial least square discriminant analysis (OPLS-DA), and s-plot score analysis. The molecular weight, retention time, and MS/MS fragmentation ion characteristics of the different compounds were compared by using Xcalibur (version 4.3), and the structures were identified and elucidated in HMDB and KEGG databases. Finally, the selected endogenous compounds were input into the Metabo Analyst system to identify potential metabolic pathways related to the different compounds.

### Western Blot Analysis

Fifty mg of the liver tissue was added with the lysis buffer for the lysis on ice for 1 h, and the lysate was centrifuged at 12,000 rpm for 5 min. Then, the supernatant was taken and the protein content in it was determined by bicinchoninic acid, and the supernatant was stored at −20°C for use. The proteins were separated by sodium dodecyl sulfate-polyacrylamide gel electrophoresis and transferred onto poly(vinylidene difluoride) membranes (2 h). Tris-buffered saline with Tween 20 (TBST) containing 5% skimmed milk powder was added onto the membranes for blocking (1.5 h), and then, the blocking solution was discarded and the membranes were washed with TBST three times, with 10 min each time. The primary antibodies UGP2 (1:1,000), UGDH (1:1,000), FAS (1:1,000), and ACC (1:1,000) were added onto the membranes, and the membranes were incubated at 4°C overnight. Then, the membranes were washed with TBST three times, with 10 min each time. The membranes were incubated with the secondary antibody at room temperature for 1 h, and then, the enhanced chemiluminescence solution ECL was added onto the membranes for development after they were washed with TBST in the same way and photographed with a gel imager. GAPDH was used as the internal reference, and the gray value of each band was measured with Image J image analysis software. The ratio of each gray value to the gray value of GAPDH was the relative expression of proteins.

### Statistical Analysis

Statistical analysis was performed using SPSS software (Windows version 19.0; IBM Corp., Armonk, NY, United States). One-way ANOVA was used for the comparison between groups. It was considered that *p* < 0.05 indicated a statistically significant difference.

## Results

### Polysaccharide Content in *Schisandra chinensis* Caulis

**FIGURE 2:**
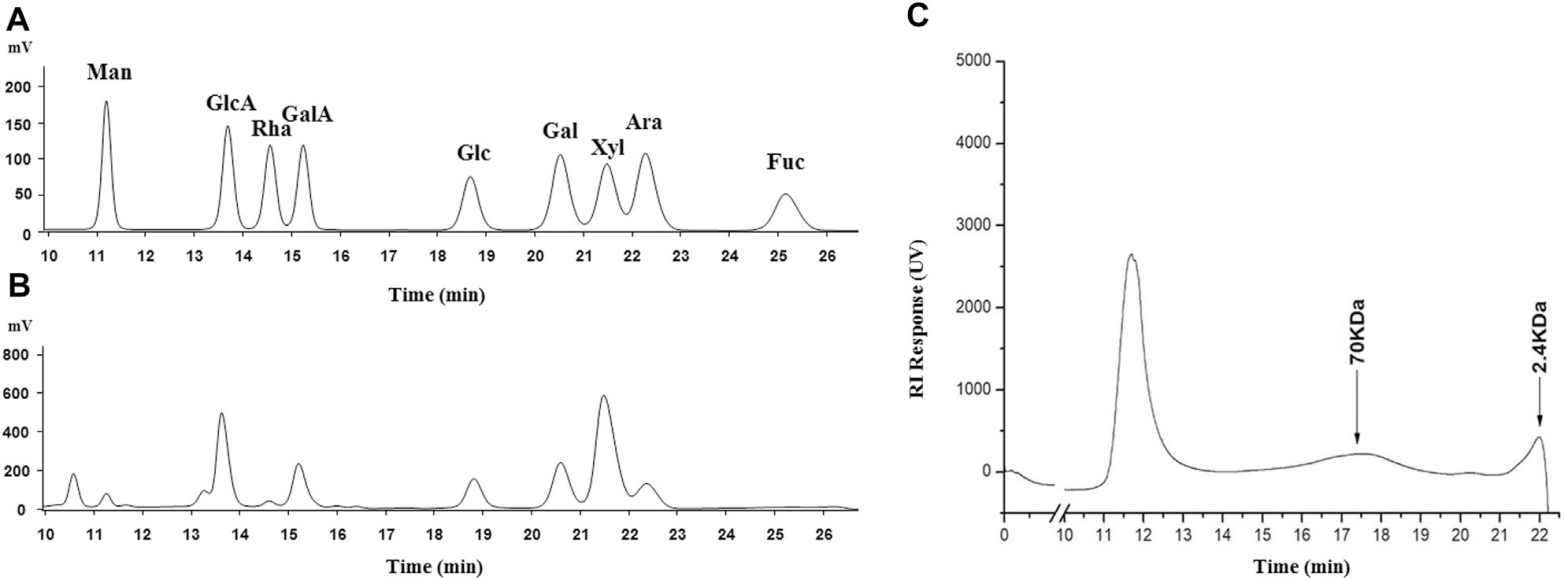
HPLC spectrograms of monosaccharide composition in SCP (**A**: reference; **B**: SCP) and molecular weight distribution of SCP **(C)**. Man, mannose; GlcA, glucuronic acid; Rha, rhamnose; GalA, galacturonic acid; Glc, glucose; Gal, galactose; Xyl, xylose; Ara, arabinose; Fuc, fructose.

After the water extraction, alcohol precipitation, and drying, 53.4 g of SCP was obtained from 1 kg of the dried *Schisandra chinensis* caulis, with a yield of 5.34%. The total carbohydrate content of SCP was 32.7%. As shown in Figure 2, the molecular weight of SCP was mainly from two fragments (peaks 2 and 3), and the relative molecular weight was 70 and 2.4 kDa, respectively. The monosaccharide contents in SCP are shown in Table 1.

**TABLE 1:** Chemical properties of SCP.

Monosaccharide composition (mol%)								
Man	GlcA	Rha	GalA	Glc	Gal	Xyl	Ara	Fuc
2.80	18.78	2.05	10.60	9.03	13.03	34.23	8.22	1.25

Man, mannose; GlcA, glucuronic acid; Rha, rhamnose; GalA, galacturonic acid; Glc, glucose; Gal, galactose; Xyl, xylose; Ara, arabinose; Fuc, fructose.

### Histomorphological Observation

**FIGURE 3:**
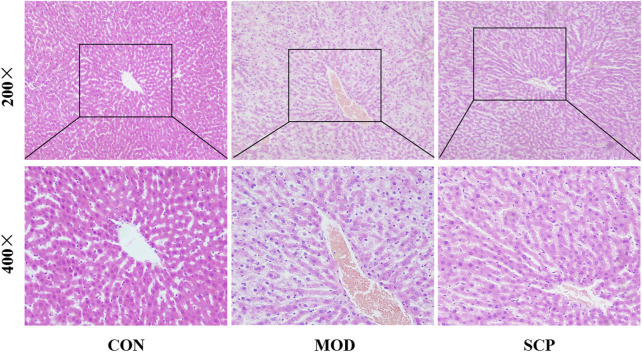
Effects of SCP on histomorphological changes in the live tissue of mice.

In the CON group, the liver tissue structure of rats was normal and the lobule structure was clear. In the MOD group, the fatty degeneration of the hepatocyte tissue of rats was obvious, accompanied with the local punctate necrosis of hepatocytes, indicating that HFD could cause a severe liver injury in rats. Compared with the MOD group, the fatty degeneration of hepatocytes in the SCP group was alleviated, the blood sinus cavity returned to normal, and the local necrosis was alleviated, indicating that SPC should have a protective effect against NAFLD caused by HFD (Figure 3).

### Effects of *Schisandra chinensis* Caulis Polysaccharides on Biochemical Indexes of Nonalcoholic Fatty Liver Disease Rats

**FIGURE 4:**
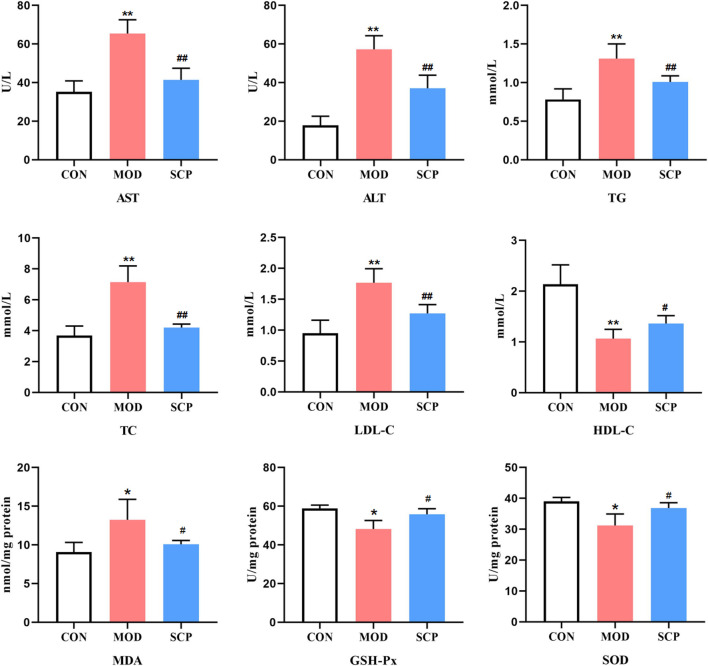
Effects of SCP on the AST, ALT, TG, TC, LDL-C, and HDL-C levels in serum, the MDA level, and GSH-Px and SOD activities in the liver tissue. All the values were expressed as means ± standard deviation; compared with the CON group, *p < 0.05, **p < 0.01; compared with the MOD group, ^#^ *p* < 0.05, ^##^ *p* < 0.01.

Compared with those in the CON group, the levels of AST, ALT, TG, TC, and LDL-C in serum and MDA in the liver were significantly increased (*p* < 0.05, *p* < 0.01), while those of HDL-C in serum and GSH-Px and SOD in the liver were significantly decreased (*p* < 0.05, *p* < 0.01) in the MOD group; compared with those in the MOD group, the levels of AST, ALT, TG, TC, and LDL-C in serum and MDA in the liver were significantly decreased (*p* < 0.05, *p* < 0.01), and those of HDL-C in serum and GSH-Px and SOD in the liver were significantly increased (*p* < 0.05) in the SCP group (Figure 4).

### Metabonomic Analysis

**FIGURE 5:**
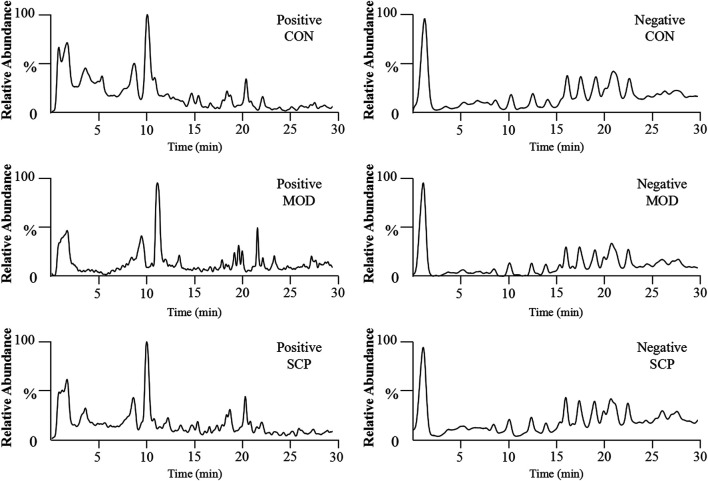
BPI chromatograms of serum samples obtained from the negative and positive ion UHPLC-Q-Orbitrap-MS analyses.

**FIGURE 6:**
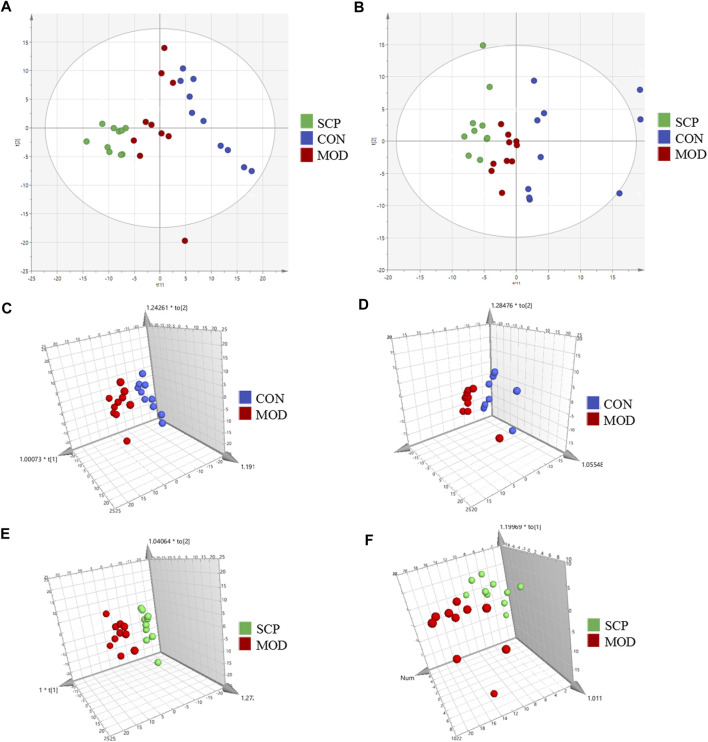
PCA score plots of the CON, MOD, and SCP groups in the positive mode **(A)** and negative mode **(B)**; OPLS-DA score plots of the CON and MOD groups in the positive mode **(C)** and negative mode **(D)**; OPLS-DA score plots of the MOD and SCP groups in the positive mode **(E)** and negative mode **(F)**.

Figure 5 shows the base peak intensity (BPI) chromatograms of representative samples in the positive and negative ion modes. Based on the UHPLC-Q-Qrbitrap-MS/MS data, the potential metabolites in the serum samples of rats in the CON, MOD, and SCP groups were analyzed by PCA. PCA, a commonly used unsupervised multivariate data analysis method, is considered to reduce the number of dimensions with a minimum information loss and show the characteristics of each sample. As shown in the PCA score plots (Figures 6A,B), the scattering points of samples in the CON, MOD, and SCP groups showed an obvious separation in the positive and negative ion modes. Then, supervised OPLS discriminant analysis was used to maximize the difference of metabolites among the three groups and detect metabolites in biological samples. The metabolites in the blood of rats in the different groups were well separated, with obvious differences, indicating that HFD and SCP may induce significant changes in related components (Figures 6C–F).

**FIGURE 7:**
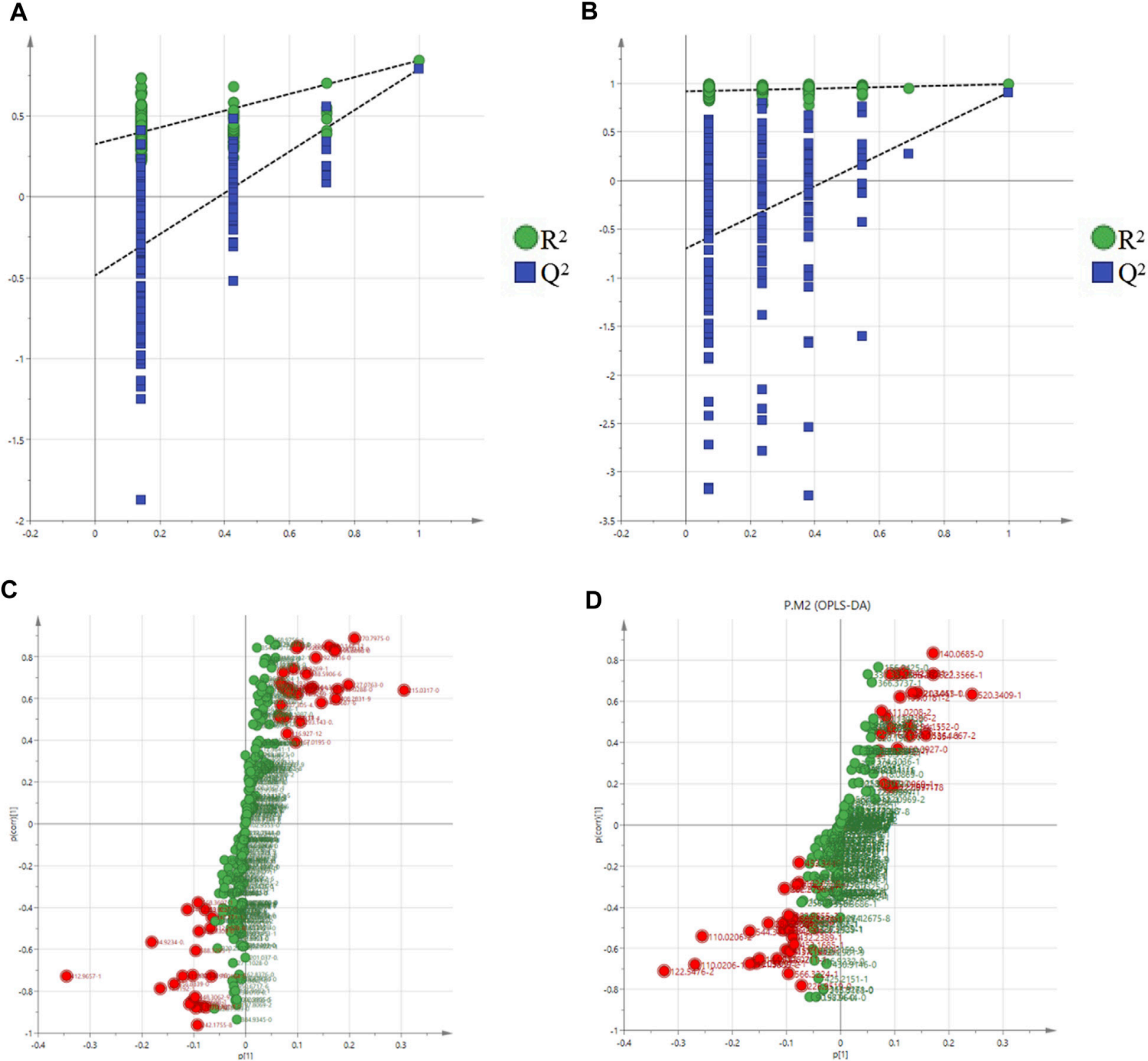
Permutation test of the OPLS-DA model in the positive mode **(A)** and negative mode **(B)**; S-plot in the positive mode **(C)** and negative mode **(D)**.

The validity of the model was verified by 100 iterations of the permutation test. The R^2^ (cumulative) and Q^2^ (cumulative) values of all permutations on the left were lower than those of the original point on the right, and the intercept of the blue regression line of the *R* ^2^ point was negative, indicating that the original model was valid (Figures 7A,B). Further S-plot analysis was performed, and compounds with an evaluation parameter of VIP > 1 were regarded as potential biomarkers based on the VIP in the projection of the OPLS-DA model (Figures 7C,D).

**FIGURE 8:**
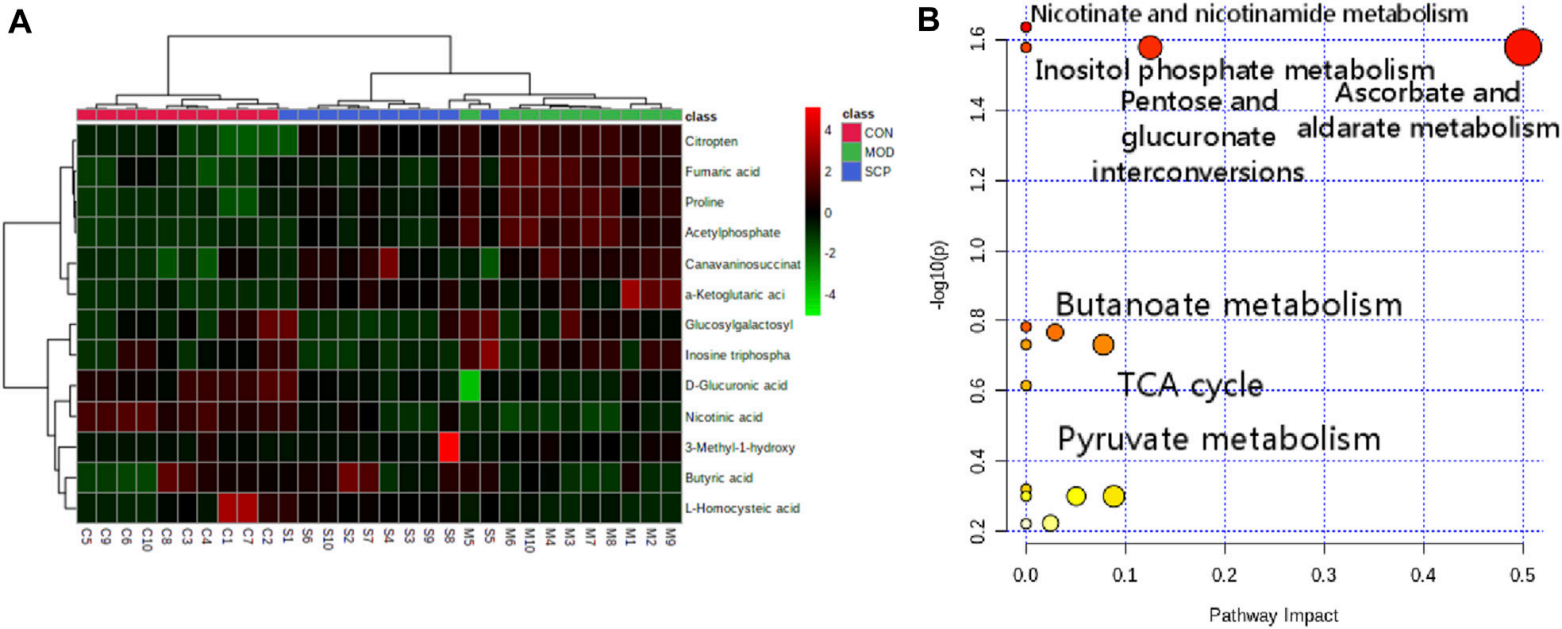
Heat map of potential biomarker changes **(A)** and pathways in SCP-treated rats **(B)**.

The accurate molecular mass and fragmentation ion characteristics (quality error <5 ppm) of the different metabolites were aligned with those in the human metabolism database (HMDB), lipidomics Gateway, and KEGG and PubChem databases, and 13 different metabolites among the groups were screened out (Table 2). The clustering analysis showed that compared with those in the CON group, the contents of citric acid, fumaric acid, proline, α-ketopentadic acid, sabouramide succinic acid, and acetylphosphate in the MOD group were significantly increased but significantly decreased after the intervention of SCP. Compared with those in the CON group, the contents of D-glucuronic acid, nicotinic acid, and butyric acid in the MOD group were significantly decreased, while those in the SCP group were significantly increased (Figure 8A). The pathway enrichment analysis of these 13 different metabolites showed that the metabolic differences were mainly in the metabolism of ascorbic acid and uronic acid, the mutual transformation of pentose and glucuronide, the metabolism of nicotinic acid and nicotinamide, the tricarboxylic acid cycle, the metabolism of butyric acid, and the metabolism of inositol phosphate (Figure 8B).

**TABLE 2:** Information of different metabolites among groups.

Mode	Time	*m/z*	Formula	VIP	Compound name	Trend		Related pathway
						C-M	M-S	
N	0.68	193.0351	C_6_H_10_O_7_	2.74	D-Glucuronic acid	↓	↑	Ascorbate and aldarate metabolism
	18.47	114.0562	C_5_H_9_NO_2_	2.62	Proline	↑	↓	Arginine and proline metabolism
	21.71	205.0514	C_11_H_10_O_4_	1.72	Citropten	↑	↓	TCA cycle
	0.75	291.0587	C_9_H_16_N_4_O_7_	1.67	Canavaninosuccinate	↑	↓	TCA cycle
	15.57	115.0029	C_4_H_4_O_4_	1.47	Fumaric acid	↑	↓	TCA cycle
	0.73	87.0469	C_4_H_8_O_2_	1.25	Butyric acid	↓	↑	Butanoate metabolism
	4.63	485.1904	C_18_H_34_N_2_O_13_	1.10	Glucosylgalactosyl hydroxylysine	↑	↓	Amino acid metabolism
	8.51	510.1124	C_17_H_29_N_4_O_8_P_2_S	1.07	3-Methyl-1-hydroxybutyl-ThPP	↑	↓	Valine, leucine, and isoleucine degradation
P	21.33	147.0299	C_5_H_6_O_5_	2.05	α-Ketoglutaric acid	↑	↓	TCA cycle
	21.53	140.9932	C_2_H_5_O_5_P	1.52	Acetylphosphate	↑	↓	Pyruvate metabolism
	13.44	508.9845	C_10_H_15_N_4_O_14_P_3_	1.46	Inosine triphosphate	↑	↓	Purine metabolism
	16.15	124.0969	C_6_H_5_NO_2_	1.34	Nicotinic acid	↓	↑	Nicotinate and nicotinamide metabolism
	21.46	184.0257	C_4_H_9_NO_5_S	1.32	L-Homocysteic acid	↓	↑	Aminoacyl-tRNA biosynthesis

### Metabolomic Validation

The analysis of the function and pathway enrichment was performed with metaboanalysis network tools, and the obtained pathways were further aligned through the KEGG database to find UDP-glucose pyrophosphorylase (UGP2), UDP glucose 6-dehydrogenase (UGDH), acetyl CoA carboxylase (ACC), and fatty acid synthase (FAS) related to the metabolic pathways. Then, western blot was used to detect their expressions in the liver tissue of rats.

**FIGURE 9:**
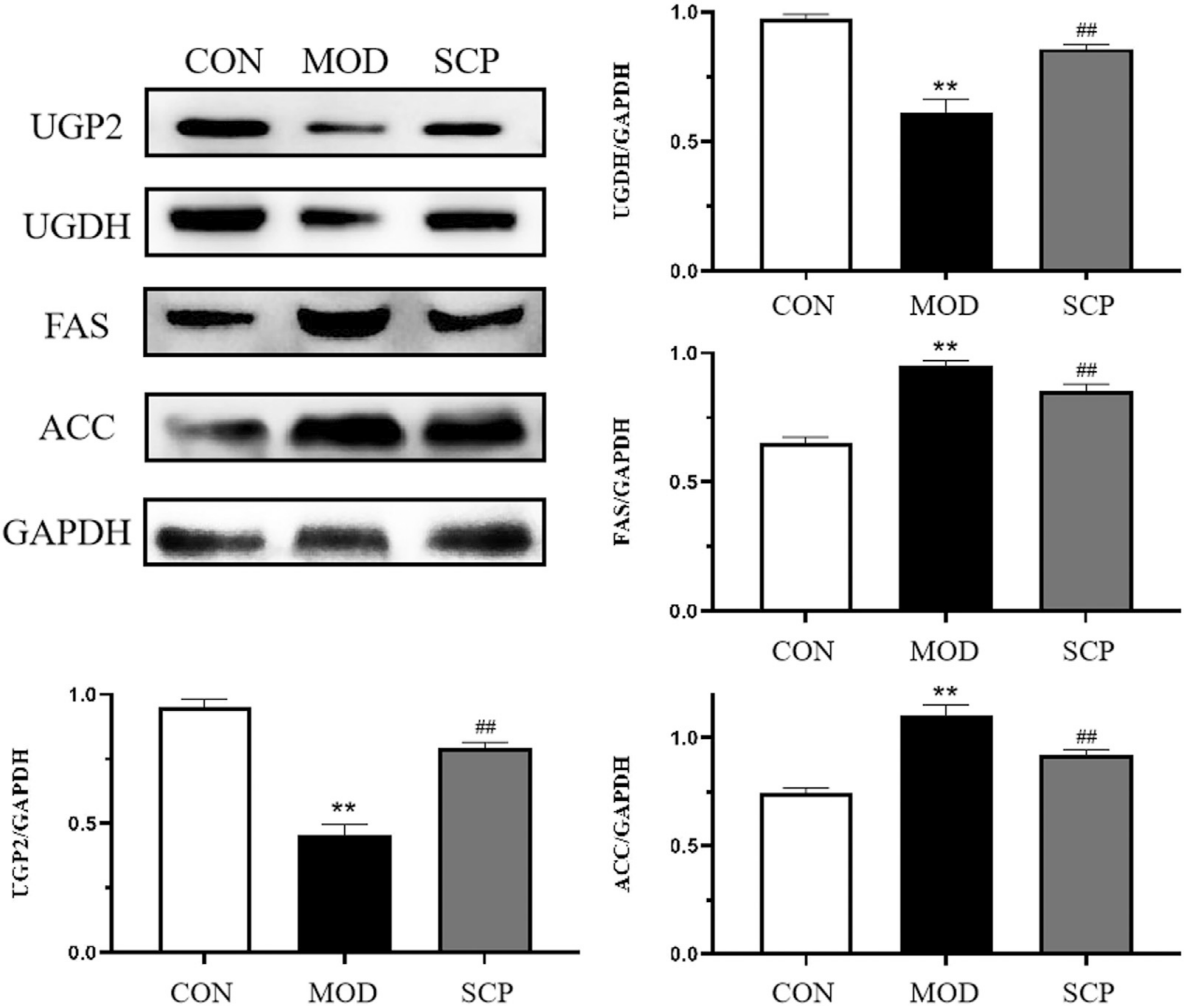
Effects of SCP on the expression of UGP2, UGDH, FAS, ACC, and GAPDH proteins in the liver tissue. All the values were expressed as means ± standard deviation. Compared with the CON group, *p < 0.05, **p < 0.01; compared with the MOD group, ^#^ *p* < 0.05, ^##^ *p* < 0.01.

As shown in Figure 9, compared with those in the CON group, the levels of UGP2 and UGDH in the liver tissue of rats were significantly decreased (*p* < 0.01), and those of FAS and ACC were significantly increased (*p* < 0.01) in the MOD group; compared with those in the MOD group, the levels of UGP2 and UGDH in the liver tissue of rats were significantly increased (*p* < 0.01), and those of FAS and ACC were significantly decreased (*p* < 0.01) in the SCP group.

## Discussion

NAFLD is one of the most important causes of liver diseases, with a global prevalence rate of about 25% (Araújo et al., 2018), and may become the most important inducing cause of advanced liver diseases such as hepatocellular carcinoma in the next few decades (Younossi et al., 2017). At present, it is widely believed that the pathogenesis of NAFLD mainly includes insulin resistance, glucose metabolism disorder, metabolic syndrome, and the abnormal lipid metabolism (Cobbina and Akhlaghi, 2017; Goedeke et al., 2018). In this study, UHPLC-Q-Orbitrap-MS/MS, a metabonomics-based method, was used to study the endogenous metabolites of small molecules in the serum of NAFLD rats after the intervention of SCP.

The enrichment analysis of metabolic pathways showed that the pathways most related to the different metabolites mainly included the metabolism of ascorbic acid and uronic acid and the transformation of pentose and glucuronic acid. These two metabolic pathways are involved in the production of D-glucose-1-phosphate, UDP-glucose, D-glucuronic acid, and pyruvate from the downstream D-glucose after the intake of sugar and nutrients by organisms. It has been reported that one of the causes of NAFLD is insulin resistance, characterized by the decreased utilization of glucose (Chen et al., 2016; Gastaldelli, 2017; Watt et al., 2019). In this study, the content of D-glucuronide in the serum of NAFLD rats was significantly lower than that in the CON group, indicating the abnormality of these two related metabolic pathways and the possibility of insulin resistance occurrence, while the content of D-glucuronide in the serum of NAFLD rats in the SCP group was increased, indicating that SCP may play a therapeutic role in NAFLD by regulating the D-glucuronide-related metabolic pathway. In order to verify this hypothesis, we first searched for the two pathways in the KEGG database, and it was found that both UGP2 and UGDH participated in the above metabolic process by participating in the mutual transformation of D-glucose 1-phosphate and UDP-glucose to produce D-glucuronic acid. It has been pointed out that UGP2, a key enzyme in glycogen biosynthesis (Looft et al., 2000; Mohammad et al., 2012), has been demonstrated to be related to the occurrence and development of a variety of cancers (Tan et al., 2014; Wang et al., 2018; Zeng et al., 2019), including hepatocellular carcinoma (HCC) (Zhou et al., 2012). Then, the expression levels of UGP2 and UGDH in the liver tissue of rats were detected by western blot. Compared with those in the CON group, the expression levels of UGP2 and UGDH in the liver tissue of rats in the MOD group were significantly decreased, confirming our hypothesis that NAFLD could cause the abnormality in the metabolism of ascorbic acid and uronic acid as well as the transformation pathway of pentose and glucuronic acid and reduce the body's utilization of glucose, resulting in gluconeogenesis; compared with those in the MOD group, the expression levels of UGP2 and UGDH in the liver tissue of rats in the SCP group increased, indicating that SCP could play a hepatoprotective role in NAFLD rats by regulating the metabolism of ascorbic acid and uronic acid as well as the transformation pathway of pentose and glucuronic acid.

Pyruvate is produced from the diet ingested by the body through the metabolism. Acetylphosphate and nicotinic acid are found to be involved in the metabolism of pyruvate and nicotinamide among the differential metabolites. Both these metabolites can affect the production of pyruvate. In particular, nicotinic acid can treat dyslipidemia and cardiovascular diseases by affecting the metabolism of lipids (Meyers et al., 2004), help to change the abnormal fat accumulation (Ganji et al., 2004), and reduce the content of plasma triglycerides (Ganji et al., 2014). In NAFLD rats, the relative content of nicotinic acid decreased sharply, while the relative content of acetylphosphate increased sharply, suggesting that the abnormality in the metabolism of both pyruvate and nicotinic acid could occur in NAFLD, and nicotinic acid could not normally inhibit the excessive production of pyruvate by regulating the lipid metabolism, increasing the content of free fatty acids in the body. Moreover, free fatty acids are accumulated in the liver due to insulin resistance, leading to steatosis in the liver (Mccommis and Finck, 2019), which can eventually evolve into NAFLD. In the SCP group, the relative level of nicotinic acid increased and the level of acetylphosphate decreased, indicating that SCP can also play a hepatoprotective role in NAFLD rats by regulating the metabolism of pyruvate and nicotinic acid.

A lower relative expression of butyric acid was also found in NAFLD rats. Butyric acid, a short-chain fatty acid, is produced by the fermentation of resistant starch, dietary fiber, and other low-digestibility polysaccharides by microorganisms in the distal intestine and colon (Kau et al., 2011). It has been found that butyric acid can affect the occurrence and development of NAFLD by reducing inflammatory response, inhibiting insulin resistance, and weakening oxidative stress of liver mitochondria (Baumann et al., 2020). Compared with that in the MOD group, the expression level of butyric acid was increased in the SCP group, indicating that SCP may alleviate NAFLD by regulating the metabolism of butyric acid.

Mitochondria are vulnerable to various damage factors such as oxides due to their high sensitivity, leading to the occurrence of a fatty liver (Horvath and Daum, 2013). High-fat diet can induce endoplasmic reticulum stress and then cause the lipid deposition in hepatocytes and the occurrence of a fatty liver (Zhang et al., 2019). It is found that ACC and FAS play an important role in the development and treatment of NAFLD. ACC catalyzes the irreversible carboxylation of acetyl coenzyme A in mitochondria to produce malonyl coenzyme A. FAS then condenses acetyl coenzyme A and malonyl coenzyme A to generate long-chain fatty acids, resulting in the fat accumulation (Kohjima et al., 2007; Kwan et al., 2015). Butyrate and pyruvate are also involved in the metabolic pathway of acetyl CoA. In addition, it has been reported that the low expression of UGP2 is significantly correlated with the fatty acid metabolism and related to fatty acid metabolic enzymes such as FAS, so it is speculated that UGP2 may play an important role in the occurrence and development of liver diseases by regulating the metabolism of fatty acid (Hu et al., 2020). The results showed that compared with those in the CON group, the levels of ACC and FAS in the MOD group were significantly increased, indicating that the low expression of UGP2 could cause the disorder of the fatty acid metabolism to cause or develop NAFLD; compared with those in the MOD group, the levels of ACC and FAS in the SCP group were significantly decreased, indicating that SCP could alleviate the disorder of the fatty acid metabolism by regulating UGP2 to regulate ACC and FAS so as to promote the decomposition and metabolism of fat, reduce the accumulation of fat in the liver of NAFLD rats, reverse the intrahepatic steatosis of NAFLD rats, and facilitate the recovery of the liver morphology, structure, and function.

**FIGURE 10:**
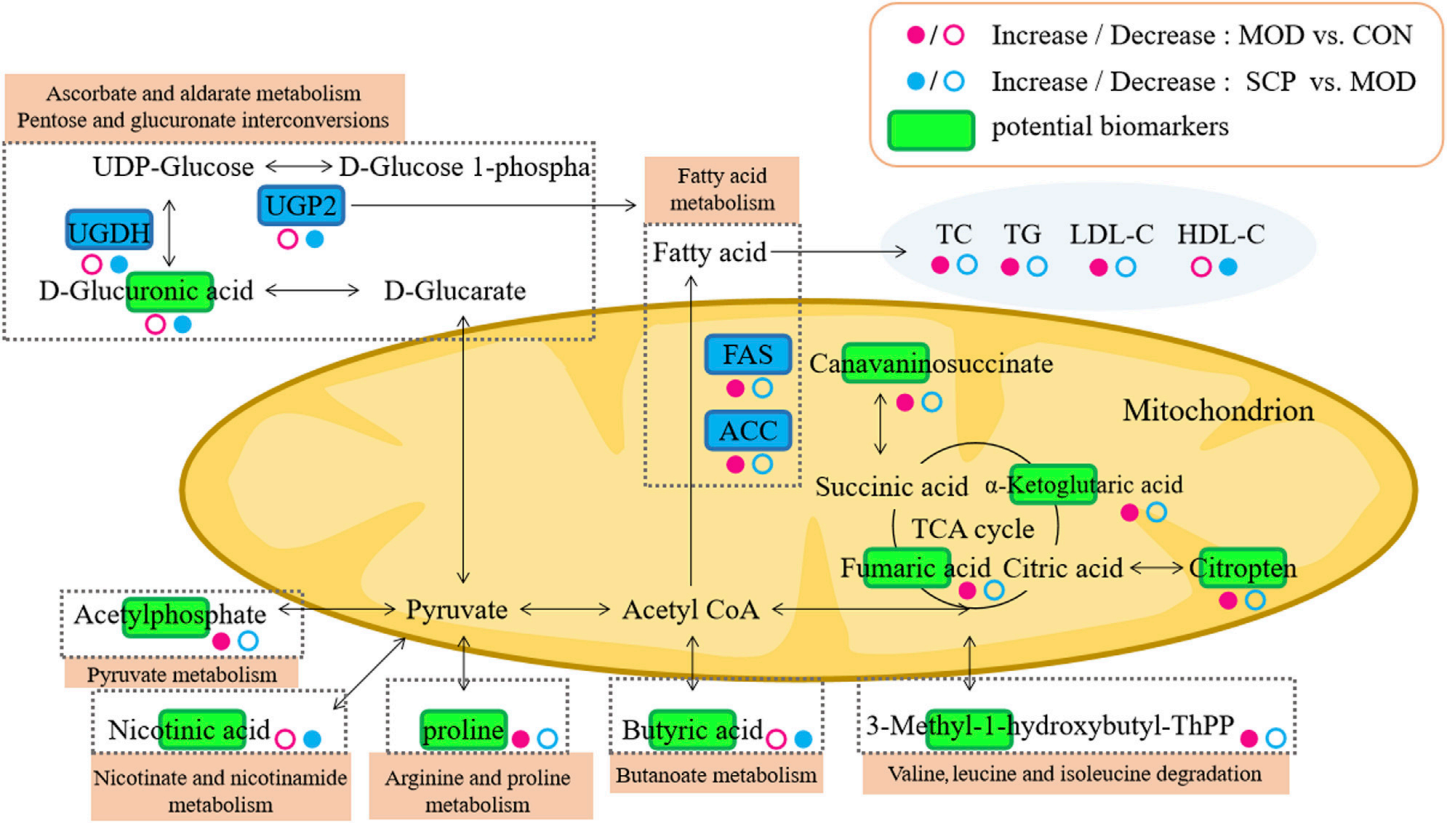
Proposed metabolic pathways regulated by SCP in NAFLD.

Pyruvate enters mitochondria for oxidative decarboxylation to form acetyl coenzyme A and finally enters the tricarboxylic acid cycle. α-Ketoglutarate, a product of oxidative deamination of glutamic acid, can participate in the tricarboxylic acid cycle with citric acid and fumaric acid together, indicating that it is an important intermediate. In this study, compared with those in the CON group, the levels of D-glucose in the serum of rats were decreased, while those of α-ketoglutarate, citric acid, and fumaric acid were increased in the MOD group, indicating that the tricarboxylic acid cycle should be inhibited and the utilization rate of glucose should be reduced, which may also be a manifestation of insulin resistance. Oxidative stress is one of the pathogenic mechanisms of NAFLD, and Kupffer cells are the main effectors responsible for the generation of reactive oxygen species (ROS), which consequently affect hepatic stellate cells (HSCs) and hepatocytes. ROS-activated HSCs undergo a phenotypic switch and deposit an excessive amount of the extracellular matrix that alters the normal liver architecture and negatively affects the liver function. Additionally, ROS stimulate necrosis and apoptosis of hepatocytes, which causes liver injury and leads to the progression of end-stage liver disease (Luangmonkong et al., 2018; Farzanegi et al., 2019). Oxidative stress can also inhibit the tricarboxylic acid cycle to cause the accumulation and increase of the above substances in the body (Langley et al., 2020). The lower levels of α-ketoglutarate, citric acid, and fumaric acid in the serum of rats in the SCP group than those in the MOD group suggest that SCP may treat NAFLD by inhibiting oxidative stress, and the results of MDA, GSH-Px, and SOD detection also confirm this. The mechanism pathways regulated by SCP in NAFLD are summarized in Figure 10.

The early diagnosis of liver steatosis lacks specific and sensitive biomarkers, and the biomarkers change significantly only in severe liver injury, so it is necessary to find more sensitive biomarkers (Chen et al., 2014). In recent years, with the continuous development of metabolomics, a large number of studies have reported the biomarkers of NAFLD, such as fatty acid amides, phosphatidylcholine, amino acids, and so forth (Caussy et al., 2019; Kim, 2021). The integration of metabolomics data and clinical information will be hopeful to discover the individual molecular characteristics in patients with NAFLD, and thereby, the patients at a risk of occurrence and progression of NAFLD will be found between patient subgroups. Our study will provide a datum support for the discovery of NAFLD biomarkers and lay a theoretical foundation for the further development and utilization of SCP.

## Data Availability

The datasets presented in this study can be found in online repositories. The names of the repository/repositories and accession number(s) can be found below: MetaboLights, MTBLS3194, https://www.ebi.ac.uk/metabolights/MTBLS3194/descriptors.

## Conclusions

Effects of SCP on plasma metabolites of NAFLD rats were studied by the metabolomics method based on UHPLC-Q-Orbitrap-MS/MS combined with multivariate analysis. A total of 13 differential metabolites were identified. The enrichment analysis found that the pathways through which SCP could play a therapeutic role in NAFLD mainly include the metabolism of ascorbic acid and uronic acid, the mutual transformation of pentose and glucuronide, the metabolism of nicotinic acid and nicotinamide, the tricarboxylic acid cycle, the metabolism of butyric acid, and the metabolism of inositol phosphate. The mechanism through which SCP could play a therapeutic role in NAFLD was confirmed by detecting the expression of the key targets in these metabolic pathways, including UGP2, UGDH, ACC, and FAS proteins. This study may provide some potential targets for the application of SCP in the treatment of NAFLD. In order to in-depth clarify the therapeutic mechanism of SCP, both *in vitro* studies on searching for immortalized cell lines and *in vivo* studies with genetic mouse models lacking the expression of the target genes should be carried out in the future.
