# Supplementary figures and images for: Study on the Hepatoprotection of Schisandra chinensis Caulis Polysaccharides in Nonalcoholic Fatty Liver Disease in Rats Based on Metabolomics
Source: Front Pharmacol. 2021 Sep 21;12:727636. doi: 10.3389/fphar.2021.727636 (PMC8490749; doi:10.3389/fphar.2021.727636)

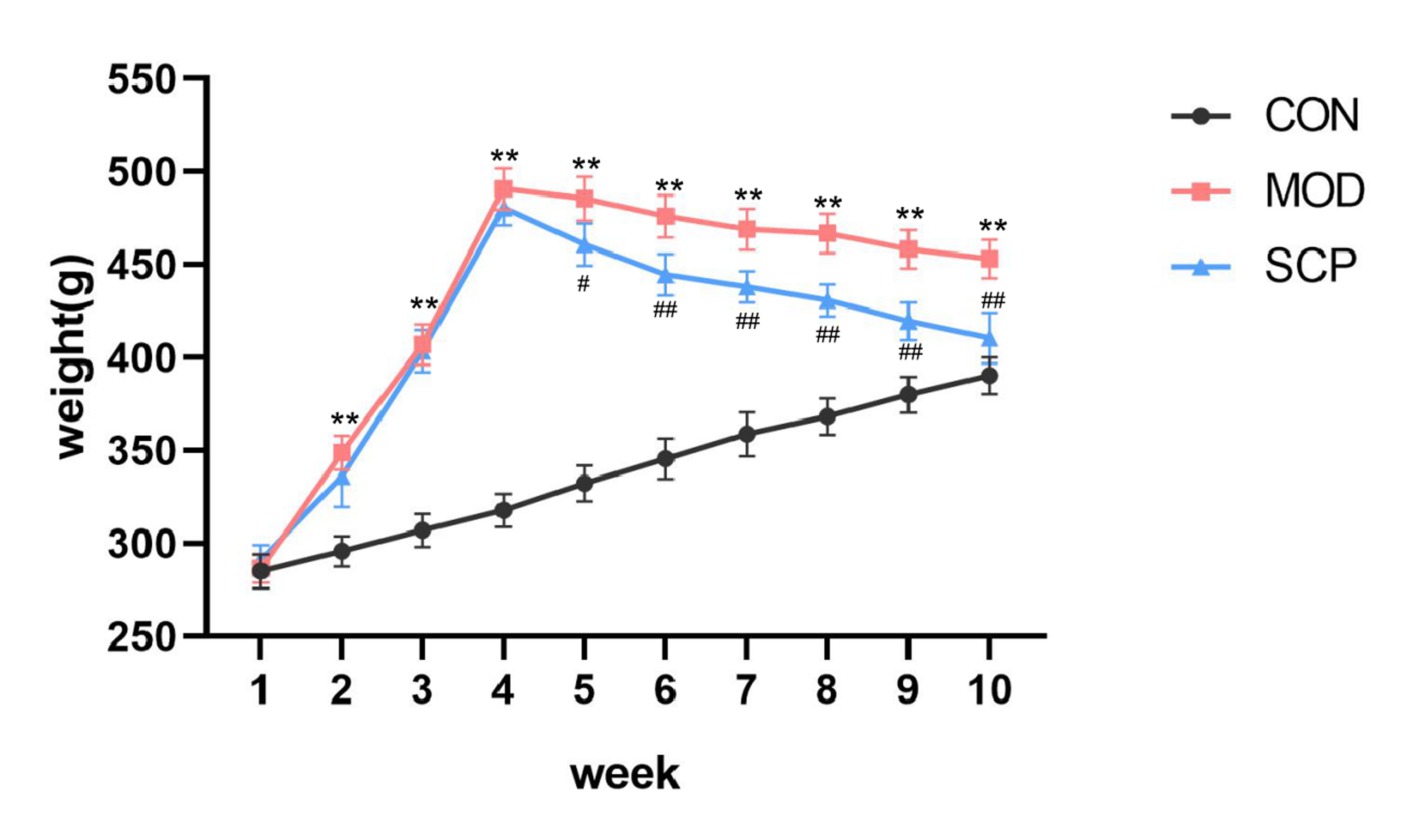

Supplement: Supplementary file 1 [file Image3.tif]

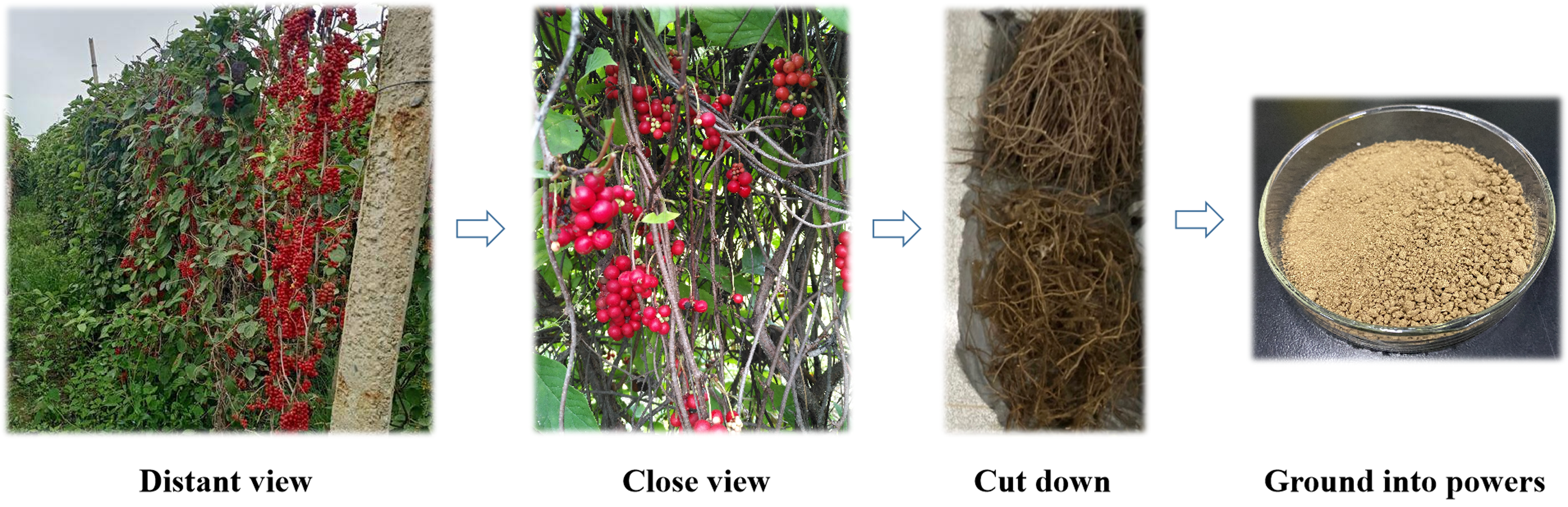

Supplement: Supplementary file 2 [file Image2.tif]

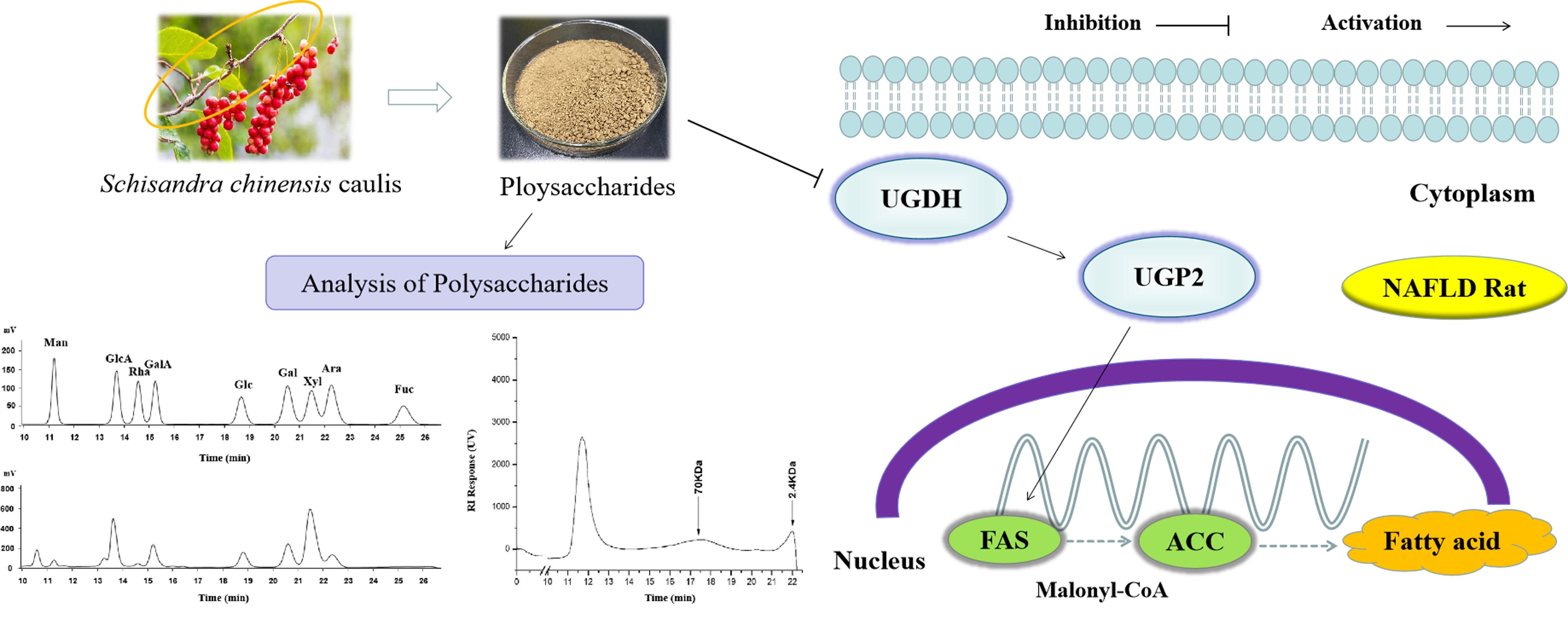

Supplement: Supplementary file 3 [file Image1.tif]
