# Supplementary material for: Study on the Hepatoprotection of Schisandra chinensis Caulis Polysaccharides in Nonalcoholic Fatty Liver Disease in Rats Based on Metabolomics
Source: Front Pharmacol. 2021 Sep 21;12:727636. doi: 10.3389/fphar.2021.727636 (PMC8490749; doi:10.3389/fphar.2021.727636)
Supplement: Supplementary file 4 [file DataSheet1.docx]

*Data article*

**Title:** *Establishment of NAFLD model induced by high fat diet in rats*

**Authors:** *Yanbo Feng, Han Li, Cong Chen, Hao Lin, Jinghui Sun*

**Affiliations:** College of Pharmacy, Beihua University, No. 3999 Binjiang East Road, Jilin 132013, China

**Contact email:** *sunjinghui2008@126.com*

**Abstract**

Nonalcoholic fatty liver disease (NAFLD) induced by high-fat diet (HFD) in rats was established by feeding with high-fat diet for 4 weeks. The body weight and NAFLD related biochemical indexes (AST, ALT, TG, TC, LDL-C and HDL-C) were detected.

**Specifications Table**

| Subject area | *Pathology* |
| --- | --- |
| More specific subject area | *Animal pathological model* |
| Type of data | *Text file and figure* |
| How data was acquired | *[UV spectrophotometer](https://fanyi.baidu.com/#en/zh/UV%20spectrophotometer) (Shanghai Macy Instrument, UV-2082H)*  *Balance (Shenyang Longteng Electronic CO. LTD, ES)* |
| Experimental factors | *After rats were given high-fat diet for 4 weeks, the level of AST, ALT, TG, TC, LDL-C and HDL-C in serum was measured. Body weight was recorded weekly.* |
| Experimental features | *Detection of AST, ALT, TG, TC, LDL-C, HDL-C and body weight* |
| Data source location |  |
| Data accessibility | *Jilin City, Jilin Province, China* |

**Value of the data**

- Nonalcoholic fatty liver disease (NAFLD) is a high incidence rate of liver disease.
- High fat diet is a classic method to establish NAFLD model.

**Establishment of NAFLD model induced by high fat diet in rats**

Nonalcoholic fatty liver disease (NAFLD) induced by high-fat diet (HFD) in rats was established by feeding with high-fat diet for 4 weeks. The body weight and NAFLD related biochemical indexes (AST, ALT, TG, TC, LDL-C and HDL-C) were detected.

1. **Experimental Design, Materials and Methods**

**1.1 Apparatus**

### UV spectrophotometer (Shanghai Macy Instrument, UV-2082H), Balance (Shenyang Longteng Electronic CO. LTD, ES).

**1.2 Chemicals and materials**

ALT, AST, TG, TC, HDL-C and LDL-C test kits (Nanjing Jiancheng Bioengineering Research Institute, Nanjing, China). The standard feed and the high-fat feed for experimental rats were provided by Changchun Yisi Experimental Animal Technology Co., Ltd. (Changchun, China). The high-fat diet contained lard (15%), sucrose (20%), cholesterol (1.2%), sodium cholate (0.2%), casein (10%), calcium hydrogen phosphate (0.6%) and basic diet (53%).

**1.3 Animal grouping and administration**

Male Wistar rats, weighing 250-300 g, were provided by Changchun Yisi Experimental Animal Technology Co., Ltd. (Changchun, China), and the certificate number was SCXK (Ji) 2019-0007. The rats were reared in separate cages in a sterile feeding room at a temperature of 18-23 ℃ and in a humidity of 40%-60%.

Thirty rats were randomly divided into control group (CON) and model group (MOD), 15 in each group. Rat in CON group were fed with the standard diet and MOD group were fed with the high-fat diet for 4 weeks. Blood samples of all the rats were collected from the abdominal aorta of anesthetized rats by the intraperitoneal injection of 25% urethane (100 mg·kg^-1^) on the 5th week. The blood samples were left standing at room temperature for 1 h, then centrifuged at 3000 r·min^-1^ to separate the serum, and the serum samples were stored at -20 ℃ for standby.

The levels of serum aspartate aminotransferase (AST), alanine aminotransferase (ALT), triglyceride (TG), total cholesterol (TC), low-density lipoprotein cholesterol (LDL-C) and high-density lipoprotein cholesterol (HDL-C), were detected by following the instructions of the test kits.

**1.4 Statistical analysis**

The statistical analysis was performed using SPSS software (Windows version 19.0; IBM Corp., Armonk, NY, USA). One-way ANOVA was used for the comparison between groups. It was considered that *p* < 0.05 indicated a statistically significant difference.

1. **Result**

**Body weight and biochemical indexes**

Figure 1 shows the weekly body weight changes of the two groups of rats. From the 1^st^ week, the body weight of MOD group were all significantly increased compared with those in CON group (*p* < 0.01).


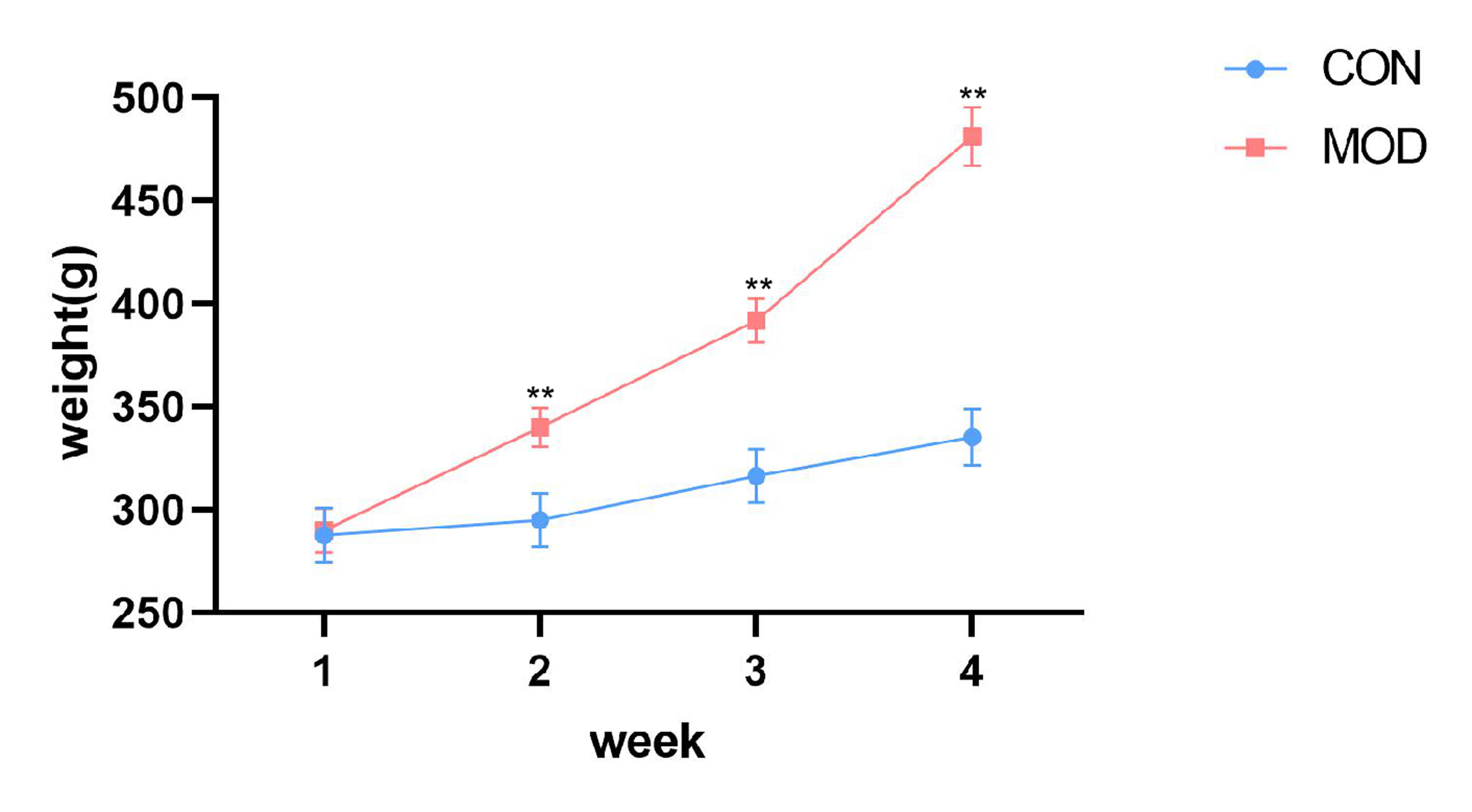


**Figure 1 Body weight of the two groups of rats**

Note: All the values were expressed as means ± standard deviation; compared with CON group, ***p* < 0.01

Compared with those in CON group, the levels of AST, ALT, TG, TC, LDL-C in serum were significantly increased (*p* < 0.01), while those of HDL-C in serum were significantly decreased (*p* < 0.01) in MOD group (Figure 2).


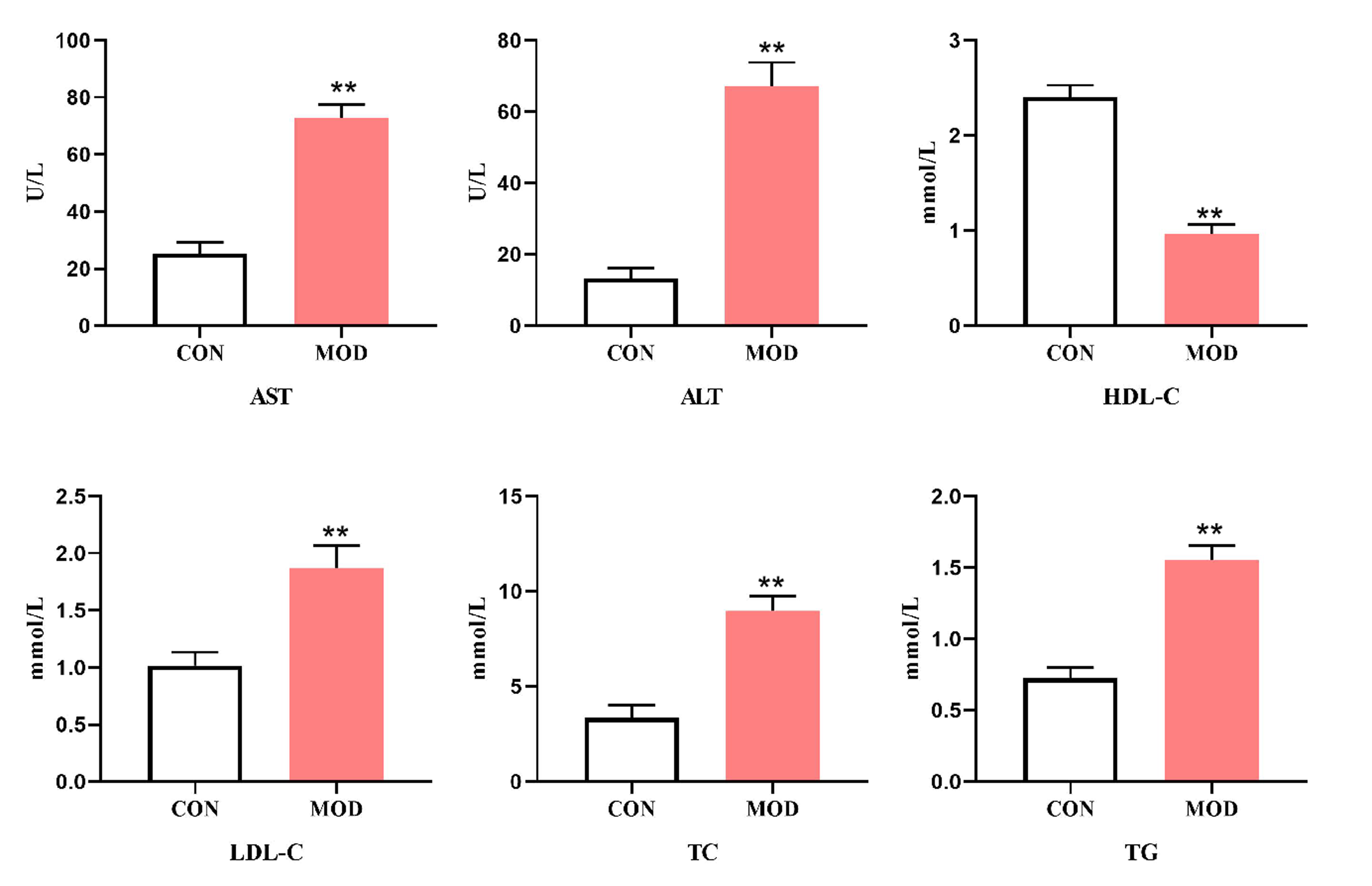


**Figure 2 AST, ALT, TG, TC, LDL-C, HDL-C levels of the two groups of rats**

Note: All the values were expressed as means ± standard deviation; compared with CON group, ***p* < 0.01
